# Efficacy of hyaluronic acid binding assay in selecting motile spermatozoa with normal morphology at high magnification

**DOI:** 10.1186/1477-7827-8-149

**Published:** 2010-12-03

**Authors:** Claudia G Petersen, Fabiana C Massaro, Ana L Mauri, Joao BA Oliveira, Ricardo LR Baruffi, Jose G Franco

**Affiliations:** 1 Department of Gynecology and Obstetrics, Botucatu Medical School Sao Paulo State University - UNESP, Botucatu, Brazil; 2 Center for Human Reproduction Professor Franco Jr., Ribeirão Preto, Brazil; 3 Paulista Center for Diagnosis, Research and Training, Ribeirão Preto, Brazil

## Abstract

**Background:**

The present study aimed to evaluate the efficacy of the hyaluronic acid (HA) binding assay in the selection of motile spermatozoa with normal morphology at high magnification (8400x).

**Methods:**

A total of 16592 prepared spermatozoa were selected and classified into two groups: Group I, spermatozoa which presented their head attached to an HA substance (HA-bound sperm), and Group II, those spermatozoa that did not attach to the HA substance (HA-unbound sperm). HA-bound and HA-unbound spermatozoa were evaluated according to the following sperm forms: 1-Normal morphology: normal nucleus (smooth, symmetric and oval configuration, length: 4.75+/-2.8 μm and width: 3.28+/-0.20 μm, no extrusion or invagination and no vacuoles occupied more than 4% of the nuclear area) as well as acrosome, post-acrosomal lamina, neck, tail, besides not presenting a cytoplasmic droplet or cytoplasm around the head; 2-Abnormalities of nuclear form (a-Large/small; b-Wide/narrow; c-Regional disorder); 3-Abnormalities of nuclear chromatin content (a-Vacuoles: occupy >4% to 50% of the nuclear area and b-Large vacuoles: occupy >50% of the nuclear area) using a high magnification (8400x) microscopy system.

**Results:**

No significant differences were obtained with respect to sperm morphological forms and the groups HA-bound and HA-unbound. 1-Normal morphology: HA-bound 2.7% and HA-unbound 2.5% (P = 0.56). 2-Abnormalities of nuclear form: a-Large/small: HA-bound 1.6% vs. HA-unbound 1.6% (P = 0.63); b-Wide/narrow: HA-bound 3.1% vs. HA-unbound 2.7% (P = 0.13); c-Regional disorders: HA-bound 4.7% vs. HA-unbound 4.4% (P = 0.34). 3. Abnormalities of nuclear chromatin content: a-Vacuoles >4% to 50%: HA-bound 72.2% vs. HA-unbound 72.5% (P = 0.74); b-Large vacuoles: HA-bound 15.7% vs. HA-unbound 16.3% (P = 0.36).

**Conclusions:**

The findings suggest that HA binding assay has limited efficacy in selecting motile spermatozoa with normal morphology at high magnification.

## Background

Up to now, different methodologies to select sperm have been described in the hope of selecting a viable sperm without - or with a low level of - DNA damage. Jakab et al. [1] was the first group that reported the use of a hyaluronic acid (HA) assay as a method to select a healthy sperm for use with ICSI. HA is a linear polysaccharide present in the extracellular matrix of cumulus oophorus around the oocyte that seems to play an important role in natural human fertilization. The use of this polysaccharide is based on the theory that hyaluronan is a major constituent of the cumulus oophorous matrix and may play a critical role in the selection of mature, functionally competent spermatozoa during *in vivo *fertilization. The principles of this assay are: (1) the expression of the protein HspA2, which indicates sperm maturation; (2) cytoplasmic membrane remodeling, which is responsible for the formation of sperm binding sites for the zona pellucida of oocytes and for HA binding sites. The Jakab group [1] suggested that immature spermatozoa present low HspA2 levels, fail to undergo cytoplasmic membrane remodeling and consequently are unable to bind to HA.

Previous studies on sperm surface markers have demonstrated that HA-bound spermatozoa are mature and devoid of cytoplasmic retention, persistent histones, apoptotic markers and DNA fragmentation [1-4]. In addition, a normal frequency of chromosomal aneuplodies [1], normal Tygerberg strict [5-8] and normal nucleus morphology criteria [9] have been correlated positively with HA-bound spermatozoa. It was shown that binding to hyaluronic acid seems to be related to one or more conventional and one or more functional sperm tests, indicating that spermatozoa from patients with abnormal conventional semen parameters have a higher likelihood for multiple functional abnormalities [10]. In addition, freezing and thawing seems not alter the HA-binding properties of the spermatozoa [11].

On the other hand, another method to select healthy sperm proposed by Bartoov et al. [12] consists of using an inverted microscope equipped with high power Nomarski optics enhanced by digital imaging to achieve a magnification ≥6300×, sufficiently high to select spermatozoa according to their nuclear fine morphological integrity, and much higher than the magnification used habitually by embryologists in sperm selection for the ICSI procedure (200×-400×) or even the one employed in the routine semen exam (1000×). The use of high magnification motile sperm organellar morphology examination (MSOME) has revealed that the selection of a morphologically normal sperm nucleus before ICSI is an important factor in improving ICSI fertilization, embryo development, pregnancy rates [13-19] and the chance of having a healthy normal child [20].

Given this context, the present study aimed to evaluate the efficacy of the HA binding assay as a method to improve selection of motile spermatozoa with normal morphology at high magnification.

## Methods

### Patients and sperm preparation

Semen samples were obtained by masturbation after 2-5 days abstinence from 56 selected men with mean age 37.9 ± 5.9 year who attended the infertility investigation and treatment. This study received internal Institutional Review Board approval and all patients signed an informed consent. Inclusion criteria: samples with ≥20 × 10^6 ^spermatozoa/ml, ≥50% progressive motility and ≤1.0 × 10^6 ^leukocytes according to the World Health Organization criteria [21].The mean percentage of sperm normal form by MSOME analysis was 1.8 ± 2.5%, as in our previous experience [22,23]. The liquefied fresh semen samples were prepared by using swim up method which consisted of leaving the ejaculated sperm sample to migrate up to the human tubal fluid (HTF) medium with 10% human serum albumin (10% HSA) deposited on the top of the fresh semen sample, at 2:1 proportion, for 30 minutes at the temperature of 37°C. The supernatant, with motile spermatozoa was removed, mixed with 1 ml of HTF 10%HSA and centrifuged at 1200 g for 10 minutes. The final pellet was adjusted to a concentration of 2 × 10^6 ^motile sperm/ml and used for the sperm-HA binding assay.

### Hyaluronic acid binding assay

For the determination and selection of the HA-bound spermatozoa, a PICSI dish for sperm selection (MidAtlantic Diagnosis - USA) was used. The three hyaluronan microdots existent in the PICSI dish were first hydrated by placing a 5 μl microdrop of mHTF 10% HSA on each microdot covered with mineral oil for 15 minutes. After this, 2 μl of prepared sperm was added into each microdrop covered by oil and incubated for at least 10 minutes to allow the spermatozoa to bind to the hyaluronan microdots. Following this period, two groups of selected spermatozoa were evaluated:

#### Group I

**Figure 1:**
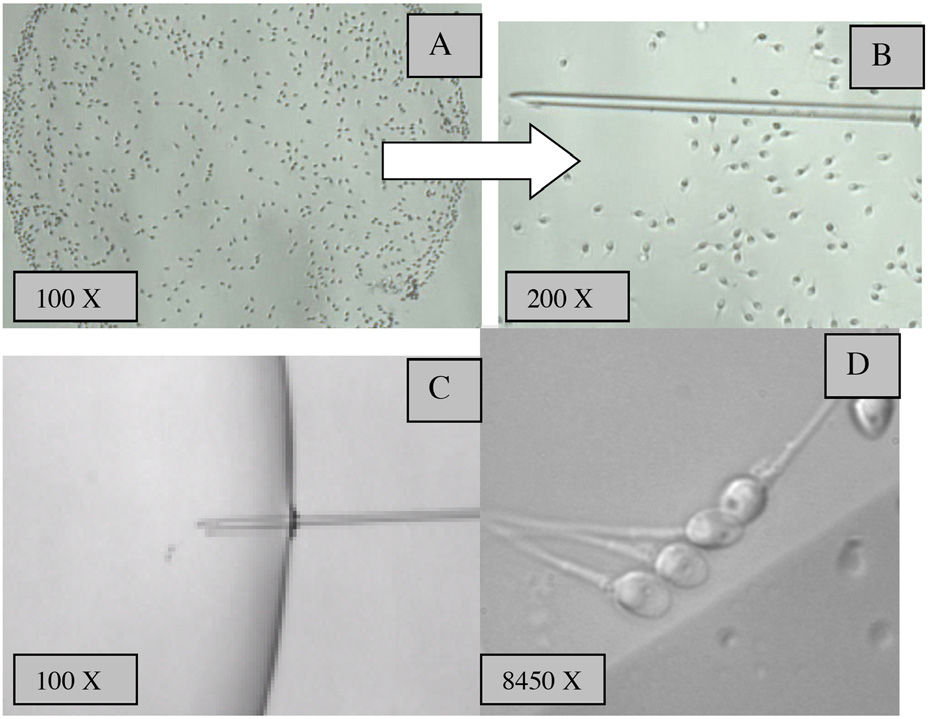
**Procedure of selecting HA bound spermatozoa and MSOME evaluation**. (A) Spermatozoa bound to the HA spot on PICSI dish and (B) HA bound spermatozoa being picked up by ICSI pipette and (C) and (D) transferred to glass dish for morphological evaluation at high magnification

(HA-bound spermatozoa): spermatozoa that presented vigorous beating, i.e. those with an increased tail cross-beat frequency and were attached by the head to the hyaluronan microdots were collected and transferred to a 3 μl microdroplet containing 7% polyvinylpyrrolidone solution (PVP - medium Irvine Scientific-USA) presented in a sterile glass dish (FluoroDishTM-Word Precision Instrument, USA), under paraffin oil (Ovoil-100, Vitrolife, Goteborg, Sweden), using an ICSI micropipette (Humagen-USA) for morphological classification by MSOME (Figure 1)

#### Group II

(HA-unbound spermatozoa): motile spermatozoa with no head attached to the hyaluronan microdots were collected and transferred to a 3 μl microdrop of 7% PVP presented in another glass dish for morphological classification by MSOME.

The entire HA procedure was performed at room temperature according to PICSI dish manufactures' guidelines (MidAtlantic diagnostic, Mount Laurel, NJ, USA), since the binding of sperm to the hyaluronan microdot is reduced at temperature above 30°C. The same embryologist, who is checked by the lab procedures in an internal lab control quality, carried out the entire HA analysis

### Classification of sperm morphology by MSOME

Both HA-bound and HA-unbound spermatozoa were analyzed at high magnification. The microdroplets were placed on a microscope with an Uplan Apo 100× oil/1.35 objective lens previously covered by a droplet of immersion oil. In this manner, suspended motile spermatozoa in the observation droplet could be examined at high magnification using an inverted microscope (Eclipse TE 2000 U Nikon, Japan) equipped with high-power differential interference contrast optics (DIC/Nomarski). The total calculated magnification was 8400× (total magnification: objective magnification = 100X magnification selector = 1.0X video coupler magnification = 1.0X calculated video magnification = 84.00). Other technician blinded to HA classification performed all sperm morphology. A total of at least 250 spermatozoa/patient were evaluated and the percentages of the following sperm forms were determined:

#### Normal spermatozoa

**Figure 2:**
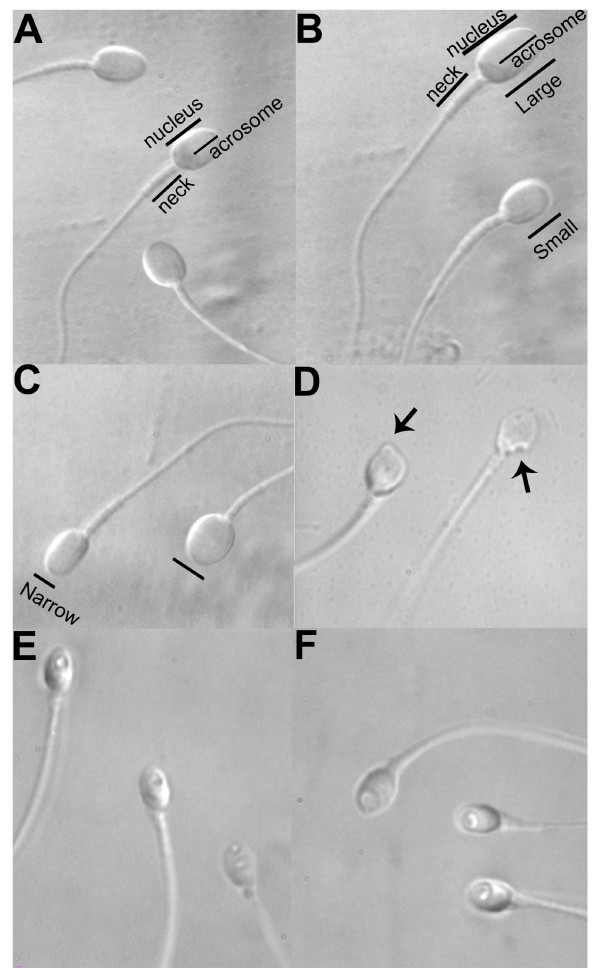
**Spermatozoa observed at high magnification (8400×)**. (A) Normal spermatozoa, (B-D)Abnormalityof nuclearform: spermatozoa with small or large oval nuclear forms (B), spermatozoa with wide or narrow nuclear forms (C), spermatozoa with regional shape abnormality of nuclear form (D), (E-F) Abnormalities in nuclear chromatin: spermatozoa with vacuoles occupying >4-50% of the nuclear area (E) spermatozoa with large nuclear vacuoles (> 50% of the nuclear area)(F).

A spermatozoon was classified as morphologically normal (Figure 2A) when it exhibited a normal nucleus as well as acrosome, post-acrosomal lamina, neck, tail, besides not presenting a cytoplasmic droplet or cytoplasm around the head [12]. For the nucleus, the morphological state was defined by the form and content of the chromatin. The criterion for normality of nuclear form was a smooth, symmetric and oval configuration. Normal means for length and width were estimated as 4.75 ± 2.8 and 3.28 ± 0.20 μm [12], respectively, whereas the form classified as abnormal presented a minimum variation of 2SD in at least one of the axes (length: ≥5.31 or ≤4.19 μm, width: >3.7 or <2.9 mm). For rapid evaluation of nuclear form, a fixed, transparent, celluloid form of sperm nucleus fitting the criteria was superimposed on the examined cell (chablon construction based on ASTM E 1951-2 [24]). In the same manner, the form of the nucleus was considered normal if no extrusion or invagination of the nuclear chromatin mass had been detected (regional abnormality of nuclear form). Chromatin content was considered normal if vacuoles occupied no more than 4% of the nuclear area. A nucleus was considered normal if both nuclear form and chromatin content were normal.

### Abnormalities of nuclear form

a-Spermatozoa with small or large oval nuclear forms (Figure 2B) - Sperm cells exhibiting an abnormal but oval nuclear shape and a morphologically normal nucleus [16], content length ≤4.19 μm or≥5.31 μm.

b-Spermatozoa with wide or narrow nuclear forms (Figure 2C) - Sperm cells with non-oval, abnormal nuclear shapes, but with normal nuclear content [16]. Width: >3.7 or <2.9 μm.

c-Spermatozoa with regional shape abnormality of nuclear form (Figure 2D) - sperm cells with an extrusion or invagination of the nuclear mass [16].

### Abnormalities of nuclear chromatin content

a-Spermatozoa with vacuoles occupying >4-50% of the nuclear area (Figure 2E)

b-Spermatozoa with large nuclear vacuoles (Figure 2F) - sperm cells with vacuoles occupying >50% of the nuclear area

Sperm cells with a severe abnormality (such as: pin, amorphous, tapered, round or multinucleated head, double tail) easily identified at low magnification (200×-400×) were not assessed in this study. The abnormalities observed at high magnification, in both form and nuclear content, also presented normal acrosome, post-acrosomal lamina, neck, tail, and did not show a cytoplasmic droplet or cytoplasm around the head. Spermatozoids that presented more than one alteration were classified as having the most severe alteration [15,16] (small/large < wide/narrow < regional shape abnormality < with vacuoles occupying >4% to 50% of the nuclear area < with vacuoles occupying >50% of the nuclear area)

### Statistical analysis

Data reported as means SD were analyzed using Instat version 3.0 (GraphPad Software, San Diego, CA, USA) on a Macintosh computer (Apple Computer In, Cupertino, CA, USA). Based on our previous experience in sperm morphology classification using high magnification [22,23,25], at least 200 motile spermatozoa per patient were selected. The percentages of sperm forms by MSOME and the HA-bound and HA-unbound spermatozoa were evaluated using Chi-square test. The significance level was set at *P* < 0.05.

## Results

The general characteristics of the men in the studied population are summarized in Table 1. From a total of 16.592 of 56 patients (± 300 spermatozoa/patient), out of a total of 5579 HA-bound spermatozoa evaluated, 2.7% presented normal morphology, 1.6% had large/small nuclear form, 3.1% showed wide/narrow nuclear form, 4.7% presented regional disorder, 72.2% presented vacuoles on four to 50% of the nuclear area and 15.7% had large vacuoles. These results did not differ statistically (*P* > 0.05) from the group of HA unbound spermatozoa (n = 11013) of which 2.5% presented normal morphology, 1.6% had large/small nuclear form, 2.7% displayed wide/narrow nuclear form, 4.4% possessed a regional disorder, 72.5% displayed vacuoles on four to 50% of the nuclear area and 16.3% had large vacuoles (Table 2).

**Table 1:** General characteristics of the men in the studied population

Characteristics	
Patients (n)	56
Age (years)	37.9 ± 5.9 (26-49)
Volume (ml)^a^	2.6 ± 1.1 (1. 0-6.0)
**Mean sperm concentration ^a^**	
Total concentration (x10^6^/ml)	82.3 ± 35.4 (24.5-150)
Spermatozoa with progressive motility (A +B) (%)	59.6 ± 15.1 (26-85)
Spermatozoa without progressive motility (%)	5.6 ± 2.0 (1-25)
Spermatozoa with no motility (%)	34.8 ± 13.13 (9-62)

Values are mean ± SD; range or % (*n*).

^a^Categorized according to World Health Organization [21].

**Table 2:** Frequency of sperm forms by MSOME classification according to hyaluronic acid (HA)bound test

Sperm form by MSOME	HA-bound Spermatozoa n = 5.579	HA-unbound Spermatozoa n = 11.013	*P*
Normal spermatozoa			
n (range)	151 (0-23)	280 (0-22)	0.56
% (range)	2.7 ± 3.8% (0%-12.9%)	2.6 ± 2.6% (0%-11%)	
Large/small spermatozoa			
n (range)	86 (0-11)	182 (0-14)	0.63
% (range)	(1.5 ± 2.2%) (0%-10.5%)	(1.7 ± 1.8%) (0%-7.4%)	
wide/narrow spermatozoa			
n (range)	173 (0-16)	295 (0-16)	0.13
% (range)	(3.1 ± 3.5%) (0%-19.3%)	(2.8 ± 2.4%) (0%-13%)	
spermatozoa with regional disorder			
n (range)	263 (0-25)	482 (0-31)	0.34
% (range)	(4.7 ± 5.1%) (0%-12.5%)	(4.3 ± 2.8%) (0%-15.5%)	
spermatozoa with vacuoles occupying >4%-50% of the nuclear area			
n (range)	4029 (0-153)	7981 (0-153)	0.74
% (range)	(72.5 ± 11.0%) (0%-89.1%)	(72.0 ± 10.30%) (0%-87%)	
spermatozoa with vacuoles occupying >50% of the nuclear area			
n (range)	877 (0-65)	1793 (0-136)	0.36
% (range)	(15.6 ± 10.8%) (0%-40.6%)	(16.5 ± 10.7%) (0-68%)	

## Discussion

In the last decade, both MSOME and the HA method have been demonstrated efficacious in selecting spermatozoa with high DNA integrity and normal morphology. Bertovitz et al. [16] have suggested that the sperm nuclear morphology, under high magnification, is one of the major parameters for sperm quality. They argued the hypothesis that vacuolization of the sperm nucleus reflects some DNA defect. In concordance with their hypothesis, Franco et al. [26] demonstrated an association between large vacuoles and the presence of DNA damage (fragmentation and denaturation) in the spermatozoa. The authors have demonstrated that spermatozoa with large vacuoles, selected at a high magnification (8400×) and directly assayed for DNA fragmentation by the Tunnel methodology, presented twice the chance of having DNA fragmentation compared to those spermatozoa with normal nuclei, i.e., without large vacuoles. These data have been confirmed by Garolla et al. [27], who have shown that the presence of nuclear vacuoles affects mitochondrial function, chromatin status, and aneuploidy rates.

With respect to the HA assay, in a consecutive series of studies on sperm surface makers, Huszar's group was the first to argue that its assay permits the selection of mature spermatozoa with no DNA damage [1-3,28,29]. In contrast to this hypothesis, Petersen et al. [30] found no correlation between the HA-binding assay (PICSI) and a low degree of DNA damage. The HA-bound spermatozoa did not differ from HA-unbound ones as to DNA fragmentation (19.6% versus 21.4%, respectively).

More recently, Parmegiani et al. [9] using a medium with HA (Sperm Slow-Medicult) to select HA-bound sperm has demonstrated an optimized ICSI outcome by favoring the selection of spermatozoa without DNA fragmentation. They studied 20 patients, and after analyzing 4000 HA-bound spermatozoa by the sperm chromatin dispersion (SCD) method, showed a reduction in rate of DNA fragmentation (5.3%) that was slightly less than one half the percentage obtained by collecting 4000 spermatozoa directly from the PVP (11%), as in a conventional ICSI procedure. In addition, Tarozzi et al. [8] have demonstrated a very low DNA fragmentation level by the Tunnel assay in both HA-bound sperm and sperm prepared by density gradient separation (1.17% and 1.59%, respectively). However, despite such low rates, their results underscore the ability of HA to select spermatozoa with higher DNA integrity. The discrepancies among these three studies [8,9,30] may be due to the different HA-binding methods used: PICSI dish, sperm slow medium and HA coated chamber. In addition, the low DNA fragmentation rate demonstrated in the Tarozzi et al. [8] study differs from the literature data [31,32], thus hindering the ability to draw any conclusion.

On the other hand, a study evaluating the variations in the structural character and stability of the nuclear chromatin in morphologically normal human spermatozoa has demonstrated that even a normally shaped human sperm nucleus can be abnormal at the molecular or ultrastructural level [33]. Recent studies reveal a positive correlation between sperm morphology, assessed by the Tygerberg strict criteria, and HA binding [5-8]. The criteria by which the morphological normality of spermatozoa can be assessed depend on the resolution power of the optical magnification system utilized. Spermatozoa appearing as morphologically normal at 1000× magnification, as in Kruger morphology, may in fact carry various structural abnormalities that can only be observed at higher optical magnification (> 6000×). Oliveira et al. [25] found a significantly higher (*P* < 0.0001) incidence, almost three-fold greater, of normal spermatozoa by the Tygerberg criteria (9.4%) than by MSOME (3.3%), and concluded that MSOME is a superior criterion for sperm morphology classification since it identifies vacuoles and chromatin abnormalities that are not found with the same precision by the Tygerberg criteria.

More specifically, aim of the present study was to evaluate the capacity of the HA binding assay (PICSI dish procedure) to select motile spermatozoa with normal morphology. In this study we found no difference in the sperm morphology between HA-bound and HA-unbound spermatozoa, when investigated by MSOME. A total of 151 out of 5579 HA-bound spermatozoa, i.e., 2.7%, demonstrated a normal morphology compared to 2.5% of the 11013 HA-unbound sperm analyzed. Up to now, only one study has attempted to analyze HA-bound spermatozoa using MSOME as the morphological sperm classification method [9]. The authors assessed 1500 HA-bound spermatozoa and found a significantly higher percentage with nuclear normality (14.5%) than among spermatozoa collected from PVP (11%). In their study, a medium containing HA (SpermSlow-Medicult) was deposited into a glass dish and after the 15-minute sperm incubation period, HA-bound sperm morphology was evaluated at high magnification. The study's difference may be due to the different HA binding methods used, the PICSI dish, sperm slow medium and also the sperm morphology criteria: normal nucleus morphology [9] and normal spermatozoa morphology [30]. Moreover, in the present study we also evaluated for the first time, specific malformation of the nucleus shape (such as: small/long; wide/narrow; extrusion/invagination) and chromatin (medium and large vacuoles), and found that HA-bound and HA-unbound spermatozoa did not differ in any of these morphologies.

On the other hand, since it was demonstrated significant positive correlation between HA binding and morphology [10], the normozoospermic samples [21] included here could have biased the outcome. In the study of Parmegiani et al. [9] 40% of their patients presented oligozoospermic semen samples, an additional fact that might have contributed for the different results. Other important point to emphasize is the possible influence of the sperm preparation on the outcome of HA binding. It is well defined in literature that semen sample preparations improve motility and morphology. Besides, the kind of semen preparation could impact the final sample quality. Different from the others papers in which no prepared sperm sample were carried out before hyaluronic binding assay, in this study we used only prepared semen samples. In addition, difference in sperm preparation between our work (swim up) and the study of Parmegiani *et al. *[9] (density gradient) could also contributed for contradictory results.

The experimental design raises other questions. HA-bound is well characterized, however the HA-unbound sperm is a mixed population, as lack of binding may occur for several different reasons. The latter fraction may also contain sperm that are in the process of binding, but yet to be bound. The distribution 2:1 (200 HA-unbound spermatozoa: 100 AH-bound), by increasing the sample of HA-unbound spermatozoid, reduces this problem but without completely eliminate it. Still, it is unclear how the study results would be influenced by the lack normal spermatozoa in same sperm samples. In addition, it is not clear how sperm morphology is relevant in hyaluronic binding assay [10]. It is possible that some spermatozoa can be mature and contain normal DNA but morphologically abnormal [26].

In conclusion, the HA binding PICSI dish assay is not efficacious at improving the selection of motile spermatozoa with normal morphology at high magnification. However the same cannot be concluded for others HA assay methods.

## Competing interests

The authors declare that they have no competing interests.

## Authors' contributions

CGP was responsible for designing and coordinating the study. All authors were responsible for data collection, data analysis, and data interpretation in the manuscript. CGP, JBAO and JF were responsible for the statistical work and for writing the manuscript. JF was responsible for reviewing the manuscript. All authors read and approved the final manuscript.
